# Inflammatory response in CF airway epithelial cells: a comparative study of modulators and wild-type CFTR rescue

**DOI:** 10.3389/fphar.2025.1657688

**Published:** 2025-12-18

**Authors:** Amal Kouadri, Camille Lyko, Carine El Hajjar, Johanna Cormenier, Kevin Kevin Gemy, Nadia Alfaidy, Mohamed Benharouga

**Affiliations:** 1 Institut National de la Santé et de la Recherche Médicale (INSERM), U1292, Laboratoire de BioSanté, Grenoble, France; 2 Commissariat à l’Energie Atomique (CEA), DSV-IRIG, Grenoble, France; 3 Université Grenoble Alpes (UGA), Grenoble, France

**Keywords:** airway inflammation, CFTR folding, CFTR protein, cystic fibrosis, pharmacological correction

## Abstract

The combination of pharmacological modulators such as lumacaftor, tezacaftor, and elexacaftor restore CFTR activity at the plasma membrane and improve lung function in patients carrying CFTR mutations such as F508del, their effects on inflammation are less clear. This study aimed to investigate whether the inflammatory response in CF airway epithelial cells depends solely on Cl^−^ transport or also on the structural integrity of the CFTR protein. We examined the effects of several CFTR modulators and wild-type CFTR overexpression on CFTR expression, trafficking, Cl^−^ channel activity, and inflammation in human CF bronchial epithelial cells. Our results demonstrate that overexpression of wild-type CFTR fully restores Cl^−^ secretion and normalizes the inflammatory response to levels observed in non-CF cells. In contrast, pharmacological correction of CFTR-F508del leads to partial recovery of Cl^−^ transport and only limited reduction of inflammation. Structural analysis revealed that corrected CFTR-F508del fails to achieve the same conformational stability as wild-type CFTR. These findings suggest that beyond ion transport, the proper folding and structural integrity of CFTR are important for regulating inflammation, potentially through interactions with other cellular proteins involved in inflammatory pathways. This work highlights the need to develop therapeutic strategies that not only restore chloride channel function but also fully correct CFTR misfolding to better control inflammation in CF.

## Introduction

Cystic fibrosis (CF) is a genetic disorder primarily affecting the lungs and digestive system (Rowe et al., 2005), caused by mutations in the CFTR gene encoding an epithelial chloride and bicarbonate channel (Kerem et al., 1989). Over 2,000 CFTR mutations have been identified (www.genet.sickkids.on.ca; www.CFTR2.org) and classified according to their effects on CFTR biosynthesis, trafficking, stability, and function (Ranganathan et al., 2017). The most common mutation, F508del, leads to CFTR misfolding and its degradation by ER-associated quality control mechanisms (Zeng et al., 2017; Benharouga et al., 2001; Sharma et al., 2004).

To correct these defects, several small-molecule modulators, including correctors (lumacaftor, tezacaftor, elexacaftor) and potentiators (ivacaftor), are now used clinically, often in combination, particularly for F508del patients (Baroni, 2025; Becq et al., 2022; Ghelani and Schneider-Futschik, 2020). Although these treatments improve lung function, the absence of proper CFTR-mediated Cl^−^ secretion continues to contribute to thick mucus, chronic inflammation, and progressive lung damage (Hisert et al., 2017a; Villate-Beitia et al., 2017; Borcherding et al., 2019). Early inflammation in CF, detectable even without infection, suggests that CFTR dysfunction may directly modulate inflammatory pathways (Villate-Beitia et al., 2017; Borcherding et al., 2019; Kirby et al., 2013).

However, studies restoring Cl^−^ secretion in CF cell models have produced conflicting results regarding inflammation (Bangel-Ruland et al., 2015; Mall and Galietta, 2015; Pesci et al., 2015; Brewington et al., 2016; Lubamba et al., 2009; Norez et al., 2009), and clinical data indicate only partial reduction (∼60%) of inflammatory responses in modulator-treated patients (De Boeck et al., 2020). Pro-inflammatory cytokines such as TNF-α and IL-17 can even enhance modulator-evoked anion secretion by increasing intracellular Cl^−^ (Rehman et al., 2024). Altogether, these findings suggest that inflammation depends not only on CFTR-mediated ion transport but also on the presence of a structurally intact CFTR capable of interacting with signaling proteins (Ramananda et al., 2024).

Recent cryo-EM studies show that correctors stabilize CFTR by filling a cavity in TMD1 and linking unstable helices, without fully repairing its tertiary defects (Fiedorczuk and Chen, 2022a). Thus, modulators may restore chloride secretion but not complete CFTR structural integrity, potentially limiting their impact on inflammation.

Here, we investigated how several CFTR modulators targeting F508del, elexacaftor, lumacaftor, tezacaftor, genistein, and miglustat, affect CFTR expression, trafficking, chloride channel activity, and the inflammatory response in human bronchial epithelial cells (HBE, CFBE, and CFBE-wt). Our findings reveal that while wt-CFTR expression fully normalizes inflammation, modulator-corrected F508del only partially reduces it, likely due to incomplete structural restoration. This supports the idea that CFTR regulates inflammation not only as an ion channel but also as a properly folded membrane protein interacting with other yet-to-be-identified partners.

## Materials and methods

### Cell culture

The healthy human bronchial epithelial cell line (16HBE14o-; HBE), the cystic fibrosis cell line (CFBE41o-; CFBE) homozygous for the F508del mutation, CFBE cells overexpressing F508del-CFTR (CFBE-dF), and CFBE cells overexpressing wild-type CFTR (CFBE-wt), provided by D. Gruenert (University of California, San Francisco, CA, United States), were grown at 37 °C in 5% carbon dioxide (CO_2_) and 95% air. The culture media were replaced every 2 days (Gruenert et al., 1995). These cell lines were grown in Eagle’s minimum essential medium containing nonessential amino acids (Gibco, United States) supplemented with 10% fetal bovine serum (FBS) (Eurobio, France) and 2 mM L-glutamine (Sigma Aldrich). CFBE-dF and CFBE-wt cell lines were maintained under selection using 5 μg mL^-1^ puromycin (Gibco). The baby hamster kidney (BHK) cell line overexpressing wt-CFTR was cultured in DMEM/F12 medium supplemented with 5% FBS. in the presence of 250 µM methotrexate (Sharma et al., 2001). All our cell lines were cultured in the absence of antibiotic and checked routinely for *mycoplasma* contamination.

### Treatment by CFTR modulators

F508del-CFTR proteins were restored to the plasma membrane by incubating cells with various correctors 24 h prior to the experiments. Genistein (50 µM), Miglustat (200 µM), Elexacaftor (VX445, 3 μM), Tezacaftor (VX661, 18 μM), Lumacaftor (VX809, 3 μM) or a combination of VX661 and VX445 (3 μM and 18 μM). The selected concentrations were based on previous *in vitro* data describing the effect of these compounds on CF airway epithelial cells (Becq et al., 2022; Sharma et al., 2001; Hardisty et al., 2021; Haggie et al., 2017; Suaud et al., 2002).

### Isolation of microsomes

Isolation of ER, Golgi, and plasma membrane-enriched microsomes from CFBE-wt and CFBE-dF, was performed using nitrogen cavitation and differential centrifugation as described (Sharma et al., 2001). Where specified, the core-glycosylated wt or mutant CFTR was eliminated from the cells during a 3-h incubation in the presence of cycloheximide (CHX, 100 μg/mL). The microsomal pellet was resuspended in HSE medium (10 mM sodium HEPES, 0.25 M sucrose, pH 7.6) and used either immediately or after being snap-frozen in liquid nitrogen.

### Limited proteolysis and glycosidase digestion

Microsomes (50–70 µg) isolated from CFBE-wt or CFBE-dF cells were exposed to trypsin at concentration of 0.05, 0.1, 0.5, or 1 mg/mL for 15 min at 4 °C in digestion buffer (phosphate-buffered saline). Proteolysis was terminated by the addition of phenylmethylsulfonyl fluoride to 1 mM, and samples were immediately denatured in 2X Laemmli sample buffer at 37 °C for 20 min.

### Electrophoresis and immunoblotting

The cells were washed twice with ice-cold phosphate buffer saline (PBS, Sigma Aldrich) and lysed at 4 °C for 20 min in PBS containing 1% Tween added with protease inhibitors (10 mM PMSF, 1 µM leupetin/pepstatin A and 1 mg/ml of iodoacetamide).

Protein concentrations were measured using the Micro BCA protein assay kit (Thermo scientific). All protein samples were in 1X final concentration of Laemmli Sample Buffer (LSB 5X: 60 mM Tris-HCl pH 6.8, 2% SDS, 10% Glycerol, 5% β-Mercaptoethanol, 0.01% Bromophenol Blue), subjected to a SDS-PAGE (6% migration gel), transferred onto nitrocellulose membranes (Bio- Rad) and probed with corresponding primary antibodies. The membranes were then probed with a secondary antibody coupled to HRP (Covalab) and the reaction was revealed using ECL (Bio-Rad) in iBright imaging system (Thermo scientific).

CFTR immunoblotting was performed with anti-CFTR monoclonal antibodies (mAb, Abcam). Wt CFTR and F508del were visualized with the mouse monoclonal M3A7 and L12B4 anti-CFTR Abs. L12B4 recognizes the region of the cytoplasmic NBD1 of CFTR (epitope within the range of amino acid positions 386 and 412), and M3A7 recognizes to the region of the cytoplasmic NBD2 of CFTR (epitope within the range of amino acid positions 1,365 and 1,395) (Sharma et al., 2001). Immunoblots, with multiple exposures, were quantified using N.I.H. ImageJ software.

### Determination of cAMP-stimulated iodide conductance

The plasma membrane cAMP-dependent halide conductance of transfected BHK, CFBE, CFBE-wt or CFBE-dF cells was determined with iodide efflux as described (Sharma et al., 2001). In brief, the chloride content was replaced with iodide by incubating the cells in loading buffer (136 mM NaI, 3 mM KNO_3_, 2 mM Ca (NO_3_)_2_, 11 mM glucose, 20 mM HEPES, pH 7.4) for 60 min at room temperature. Iodide efflux was initiated by replacing the loading buffer with efflux medium (composed of 136 mM nitrate in place of iodide). The extracellular medium was replaced every minute with efflux buffer (1 mL). After a steady-state was reached, the intracellular cAMP level was raised by agonists (10 mM forskolin, 0.2 mM CTP-cAMP, and 0.2 mM isobutylmethylxanthine) to achieve maximal phosphorylation of wt- or F508del- CFTR, and collection of the efflux medium resumed for an additional 6–9 min. The amount of iodide in each sample was determined with an iodide selective electrode (Orion).

### Cytokine secretion by sandwich ELISA

Before cell lysis, all supernatants were kept for ELISA test. Interleukins (IL) IL-1β, IL-6, IL-8, IL-17 (A, E, F) and tumor necrosis factor-alpha (TNFα) released into the culture media and present in proteins extract were assayed using a quantitative sandwich enzyme-linked immunoassay kit (PeproTech).

According to the manufacturer, the sensitivity of this assay system is less than 10 pg/mL. For ELISA assays, very low auto-fluorescence was detected in the absence of any treatment. The value of this auto-fluorescence was systematically subtracted from the experimental values.

### Statistical analysis

Differences between mean values were compared by Student’s unpaired two-tailed t-tests using SigmaStat (Jandel Scientific Software, SanRafael, CA, United States). Data are expressed as mean ± SEM, unless otherwise indicated. Significance was set at a two-tailed p value of 0.05.

## Results

### Cellular models for the investigation of inflammation response

Three cellular models were used to investigate the endogenous inflammatory response in relation to CFTR protein expression and function.

**FIGURE 1:**
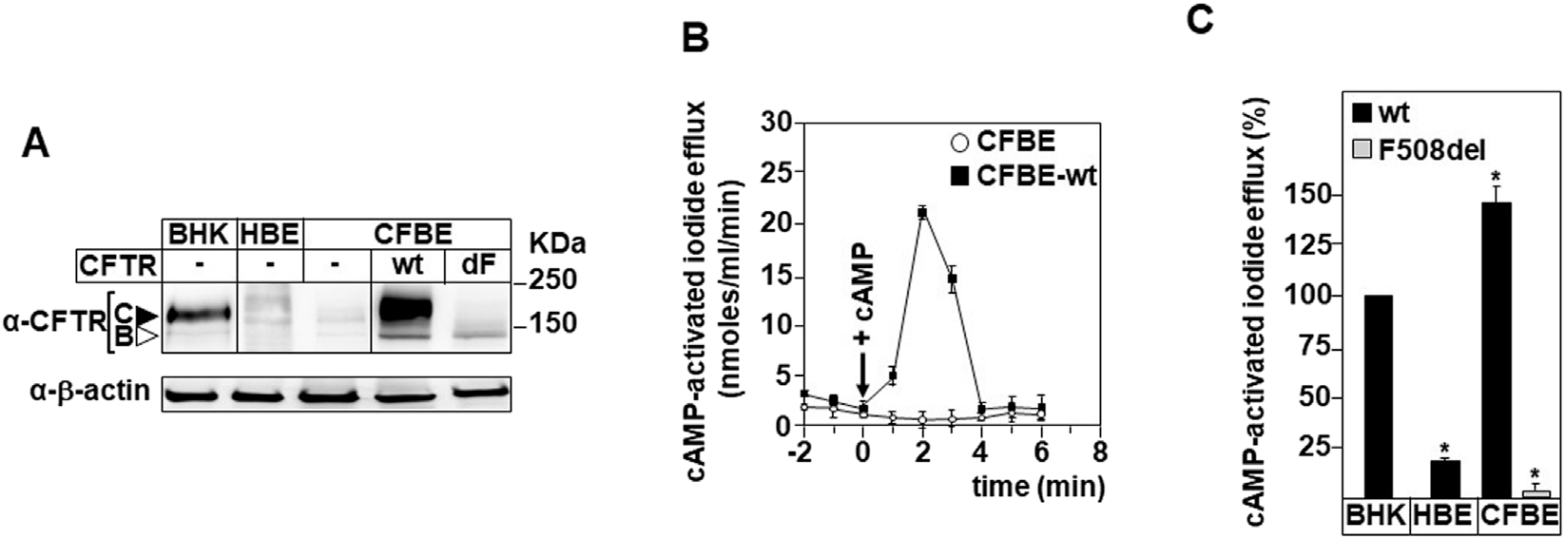
CFTR expression and function in Human Bronchial Epithelial cell models **(A)** Protein extract from BHK-21 cells stably expressing wild type CFTR, healthy (HBE), cystic fibrosis (CFBE), Human Bronchial Epithelial cells, and CFBE stably expressing wild-type (CFBE-wt) or CFTR-F508del (dF) were used in immunoblot assays to determine the expression level of CFTR protein using anti-CFTR antibody (L12B4). Black and white arrows indicate fully glycosylated mature CFTR (band C) and core-glycosylated CFTR (band B), respectively. **(B)** The iodide efflux was used as a functional assay to determine the cAMP-dependent activity of CFTR chloride channel for the indicated time. Arrow (+cAMP) indicate the time (0) where a cAMP cocktail was used to stimulate the CFTR activity. **(C)** The iodide efflux activation peak of HBE, CFBE-dF and CFBE-wt were evaluated at 2 min following the stimulation with cAMP and reported as a percentage (%) of the activation of wt-CFTR stably expressed in BHK-21 cells. Values with an asterisk are significantly different from their corresponding controls (P < 0.05). Data are expressed as mean ± SE (n = 7).

Human bronchial tissue (HBE) cells, a widely used healthy cell line and a reference model for CF (Hardisty et al., 2021), were used as controls (Figure 1A, HBE).

This latter model endogenously expresses low levels of the mutant CFTR-F508del protein (Figure 1A). To enhance the expression of CFTR-F508del while maintaining a native bronchial cell context, the CFBE-F508del (dF) model was generated (Gruenert et al., 1995) and is now widely employed to study the effects of CFTR corrector and potentiator compounds (Haggie et al., 2017) (Figure 1A, 1dF).

Additionally, CFBE cells were engineered to overexpress the wild-type CFTR protein (wt). This model enables direct comparison between the effects of pharmacological treatments and the expression of functional wt-CFTR (Figure 1A, 1wt). BHK cells were used solely as a control to compare basal CFTR expression levels in healthy and CFBE cells (Figure 1A). To evaluate CFTR function, iodide efflux was measured in the absence or presence of cAMP, which is known to activate CFTR channel gating in association with ATP (Suaud et al., 2002). Two minutes after cAMP stimulation, CFTR activity was detected in CFBE-wt cells (∼21.5 ± 0.7 nmol/mL/min), which was significantly higher than the activity measured in untransfected CFBE cells (Figure 1B).

To correlate CFTR chloride channel activity with its expression level, the iodide efflux measured at 2 min post-cAMP stimulation was expressed as a percentage of the activity measured in BHK-CFTR cells, which was arbitrarily set at 100% (Figure 1C). The results showed that HBE cells exhibited approximately 20% ± 3% of CFTR activity, while CFBE-F508del cells displayed only residual Cl^−^ channel activity (Figure 1C). In contrast, CFBE-wt cells reached ∼145% ± 5% activity, supporting a correlation between CFTR expression and functional output (Figure 1C).

### Expression and function of CFTR in the presence of correctors

**FIGURE 2:**
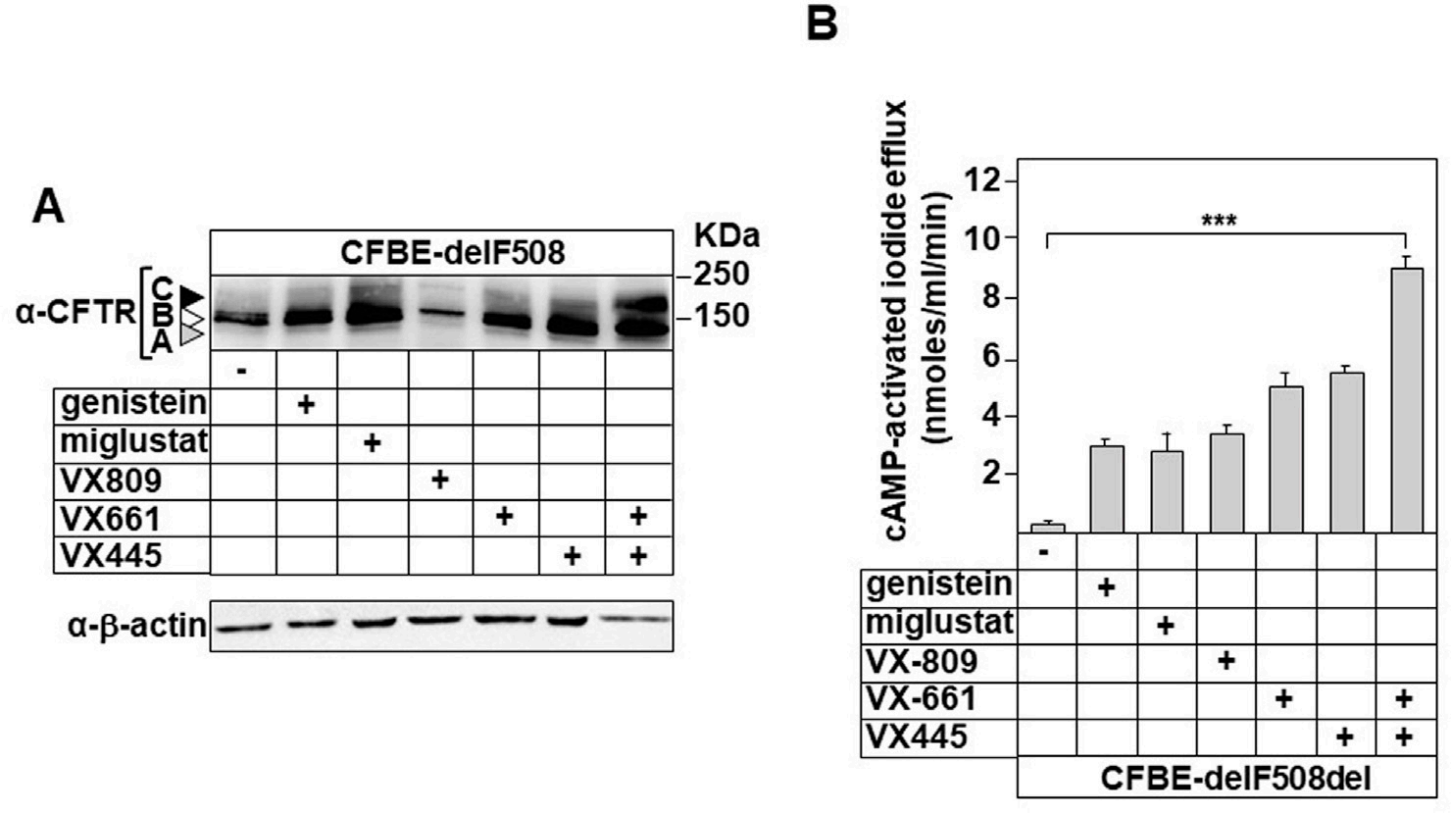
Modulation of CFTR-delF508 expression and Cl^−^ channel activity by correctors **(A)** Following 24 h treatment with tested correctors; genistein (50 µM), miglustat (200 µM), VX809 (3 μM), VX661 (18 μM), VX445 (3 μM) or VX445/VX661 (3 μM and 18 μM), protein extracts prepared form CFBE cells stably expressing CFTR-delF508 (CFBE-F508del) were subjected to immunoblot assay. Mature- (form C), immature (form B) and non- (form A) glycosylated CFTR-delF508 indicated with black, white and grey arrow, respectively, were identified using anti-CFTR antibody (L12B4). **(B)** The iodide efflux was used as a functional assay to determine the cAMP-dependent activity of CFTR chloride channel for the indicated time. The activation peak of CFBE-dF were evaluated at 2 min following the stimulation with cAMP.

The effect of correctors on CFTR-F508del expression was first evaluated by immunoblotting, detecting the appearance of bands C, B, and A corresponding to the hyperglycosylated (or mature form, ∼170 kDa), core-glycosylated (or immature form, ∼140 kDa), and non-glycosylated forms (∼135 kDa), indicated by black, white, and grey arrows, respectively (Figure 2A). Compared to the control, all tested molecules, used individually or in combination (VX661 plus VX445), induced the appearance of band C, which intensity was higher with miglustat and VX445 (Figure 2A). The combination of VX661 and VX445 dramatically increased the expression level of band C (Figure 2A, black arrow).

These molecules also enhanced the expression of band B, except for VX808 (Figure 2A, white arrow). The expression level of band C was correlated with chloride channel activity after cAMP stimulation. Our results clearly demonstrate that the VX661/VX445 combination led to a significantly higher level of activation (∼9,2 ± 0.9 nmol/mL/min) compared to individual treatments, which resulted in activation levels ranging from 3 to 5.7 nmol/mL/min.

### The inflammatory profile of CFBE-dF cells

**FIGURE 3:**
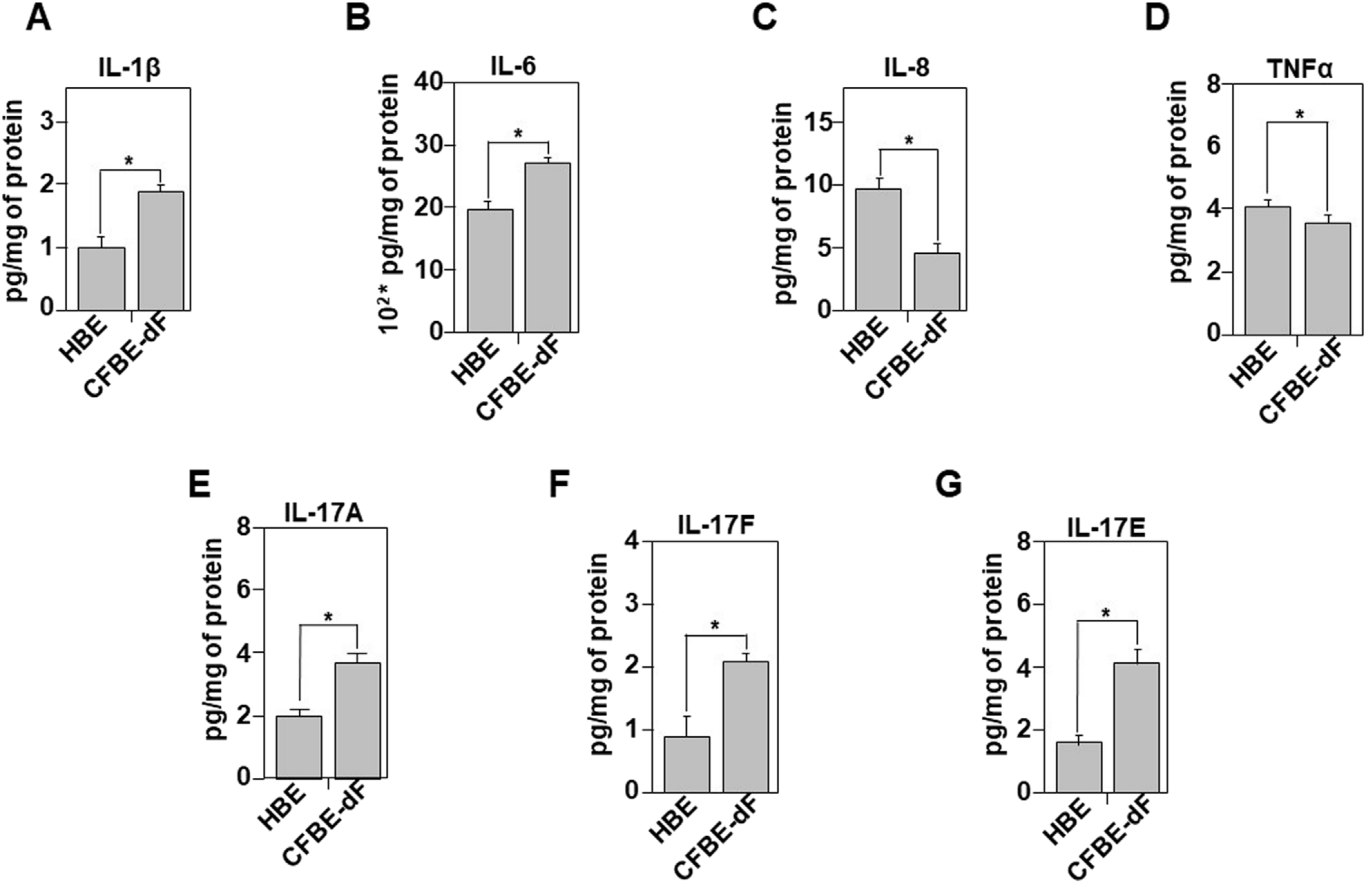
Comparative analysis of the inflammatory secretome in HBE and CFBE-dF Cells. The inflammatory profile was evaluated using a quantitative sandwich enzyme-linked immunoassay (ELISA). Measurements of IL-1β **(A)**, IL-6 **(B)**, IL-8 **(C)**, TNFα **(D)**, IL-17A **(E)**, IL-17F **(F)**, and IL-17E **(G)** were assessed in conditioned media of HBE or CFBE-F508del (CFBE-dF) cells. The results are reported in pg/mg of extracted proteins. Values with an asterisk are significantly different from their corresponding controls (P < 0.05). Data are expressed as mean ± SE (n = 7).

We used CFBE cells overexpressing the CFTR-F508del (CFBE-dF) to evaluate the effects of several correctors on CFTR expression and function. We then analyzed their steady-state inflammatory profiles in comparison to human bronchial tissue (HBE) cells (Figure 3).

Using ELISA assays, we measured both the intracellular (i) production (supplementary data) and extracellular secretion of several pro-inflammatory cytokines: IL-1β, IL-6, IL-8, IL-17A, IL-17F, IL-17E, and TNFα. Our results show that overexpression of CFTR-F508del in CFBE cells does not significantly affect either the production or secretion of the tested cytokines (Figure 3; Supplementary Figure S1).

However, compared to HBE controls, CFBE-dF cells exhibited a significant increase in both production and secretion of IL-1β, IL-6, IL-17A, IL-17F, and IL-17E (Figures 3A,B,D–G). Interestingly, although CFBE-dF cells produced high levels of IL-8, their secretion of this cytokine was significantly reduced (Figure 3C; Supplementary Figure S1). For TNFα, the decrease was observed at both the production and secretion levels (Supplementary Figure S3D).

### CFTR-wt expression improves the inflammatory state in CFBE cells

**FIGURE 4:**
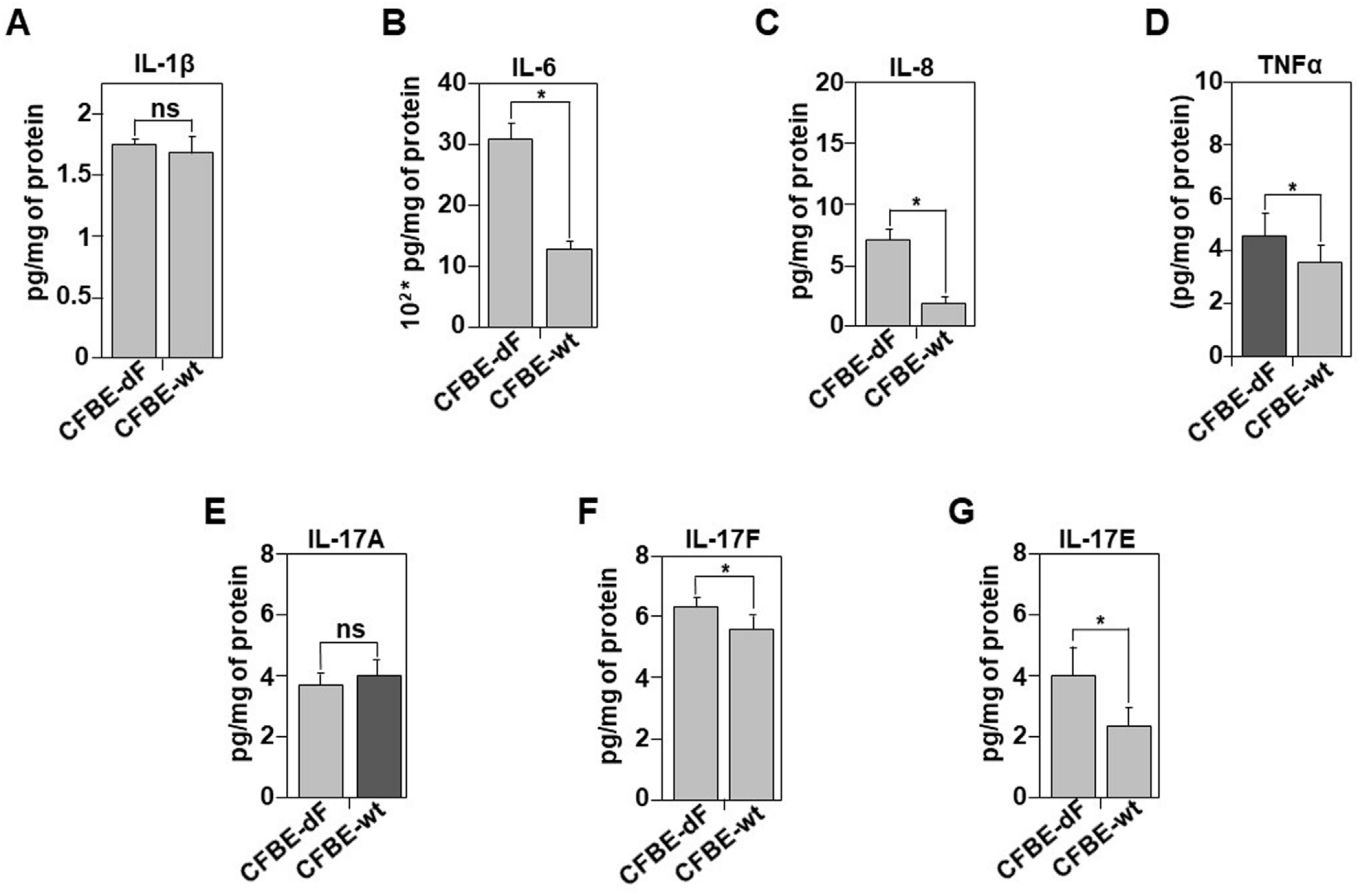
Comparative analysis of the inflammatory secretome in CFBE-dF and CFBE-wt Cells. The ELISA assay was used under the same conditions as described in Figure 3. Released culture media of CFBE-wt and CFBE-dF cells were collected and the concentration of IL-1β **(A)**, IL-6 **(B)**, IL-8 **(C)**, TNFα **(D)**, IL-17A **(E)**, IL-17F **(F)**, and IL-17E **(G)** were evaluated and expressed as pg/mg of protein extract. Values with an asterisk are significantly different from their corresponding controls (P < 0.05). Data are expressed as mean ± SE (n = 7).

To assess the corrective effect of wt-CFTR expression on the inflammatory response, we compared our results to those obtained with uncorrected CFBE cells (Figure 4).

Our analysis of secreted cytokine levels, showed no significant correction between CFBE and CFBE-wt cells regarding IL-1β and IL-17A (Figures 4A,E). IL-8 secretion remained impaired (Figure 4C) despite increased intracellular production in CFBE-wt cells (Supplementary Figure S2C). A significant reduction in IL-6 levels production (S2B) and secretion (Figure 4B), however, was observed in CFBE-wt cells. Additionally, TNFα secretion was further reduced in CFBE-wt cells compared to CFBE cells (Figure 4D), even though its production was increased (Supplementary Figure S2D). No changes were detected for IL-17A (Figure 4E), while both IL-17F and IL-17E showed a decrease in their secretion (Figures 4F,G).

### Correctors and inflammatory responses

**FIGURE 5:**
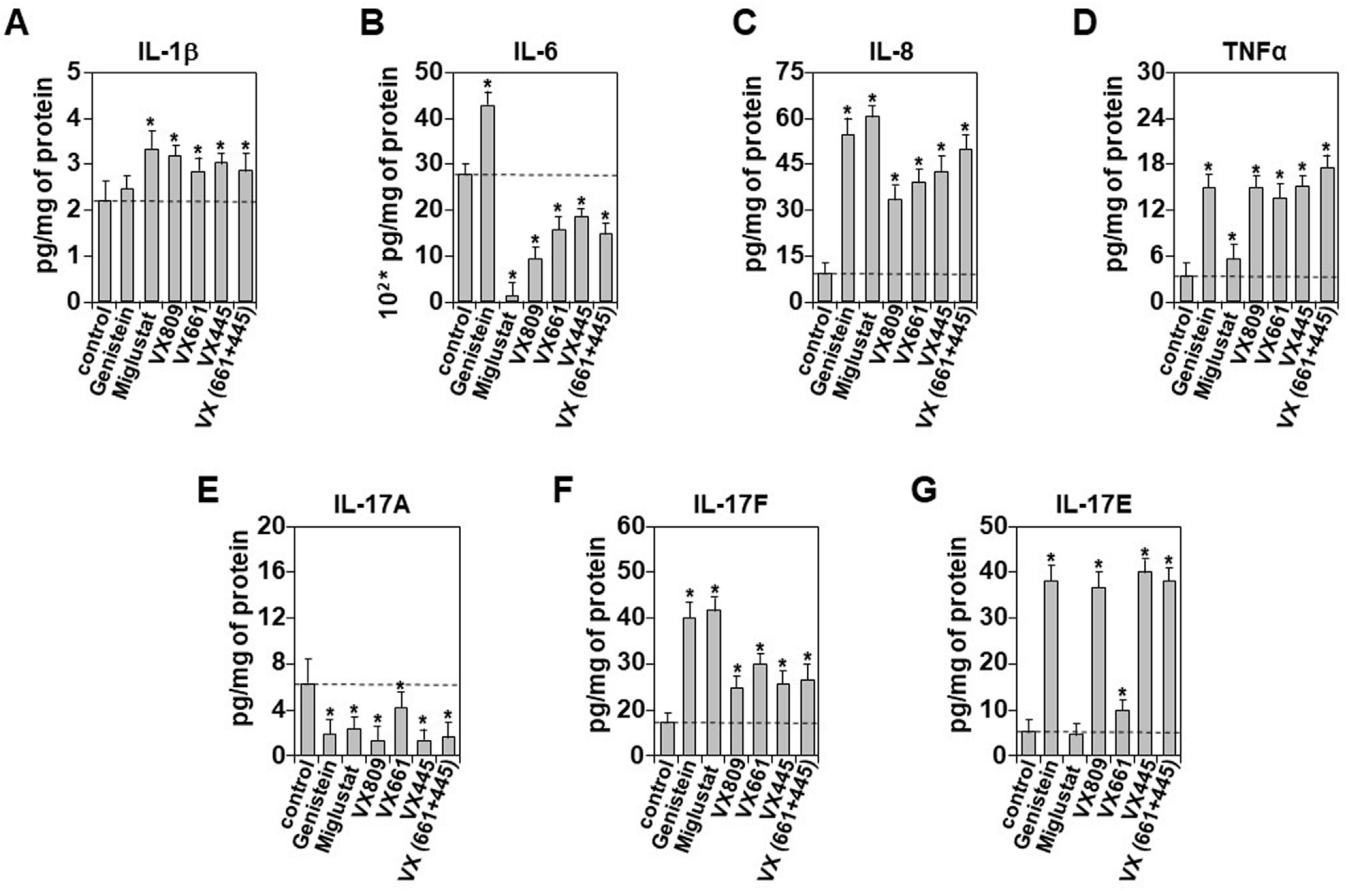
Impact of CFTR Correctors on the Inflammatory Response of CFBE-dF Cells. Following 24 h treatment of CFBE cells stably expressing CFTR-F508del (CFBE-dF) with the indicated correctors; genistein (50 µM), miglustat (200 µM), VX809 (3 μM), VX661 (18 μM), VX445 (3 μM) or VX445/VX661 (3 μM and 18 μM), release culture media were collected and the concertation of IL-1β **(A)**, IL-6 **(B)**, IL-8 **(C)**, TNFα **(D)**, IL-17A **(E)**, IL-17F **(F)**, and IL-17E **(G)** were evaluated using ELISA assays. The results are reported in pg/mg of protein extract. Values with an asterisk are significantly different from their corresponding controls (P < 0.05). Data are expressed as mean ± SE (n = 7).

In the absence of bacterial infection, the restoration of chloride ion secretion through the expression of a normal CFTR gene significantly improved the inflammatory profile of CFBE-dF cells (Figure 4). However, the effects of CFTR modulators on the production and secretion of inflammatory mediators in a non-infectious context remain poorly defined. Figure 5 present a comparative analysis of all tested molecules on the expression and secretion of IL-1β, IL-6, IL-8, TNF-α, IL-17A, IL-17F, and IL-17E.

Surprisingly, most of the tested molecules led to an increase in cytokine secretion, with the exception of IL-6 and IL-17A, for which levels were decreased (Figures 5A–G). Specifically for IL-6, all correctors except genistein significantly reduced its secretion (Figure 5B). A similar trend was observed for IL-17A (Figure 5E). These results contrast with those obtained in protein extracts (Supplementary Figure S3), where we observed a decrease in IL-8 production, except with miglustat (Supplementary Figure S3C). For IL-6, the production results were consistent with the secretion data (Supplementary Figure S3B). Taken together, our findings indicate that in the absence of bacterial infection, corrector molecules can reduce the inflammatory profile of CFBE-F508del cells by selectively downregulating the production and secretion of IL-6 and IL-17A.

### Correctors rescue but do not fully repair CFTR-delF508 folding defects

The intracellular trafficking defects and impaired chloride channel activity associated with the CFTR-F508del protein were corrected in CFBE-dF cells using two strategies: expression of the wt-CFTR protein or pharmacological treatment with correctors (Figures 1, 2). However, the effects of these two approaches on the inflammatory profile of CFBE-dF cells, in the absence of bacterial infection, were not identical. Several mechanisms could underlie this difference, including variations in the degree of CFTR protein folding.

To test this hypothesis, we performed limited proteolysis combined with immunoblot analysis to assess whether the conformation of CFTR-F508del rescued by correctors differed structurally from that of wt CFTR.

Microsomes were isolated by differential centrifugation following 3 h treatment with 100 μg/ml of cycloheximide, which decreased the core-glycosylated form by 80%–86% according to densitometric analysis (data not shown). The cleavage patterns of wt, F508del, and rescued CFTR proteins, generated by limited trypsin digestion, were analyzed by immunoblotting.

**FIGURE 6:**
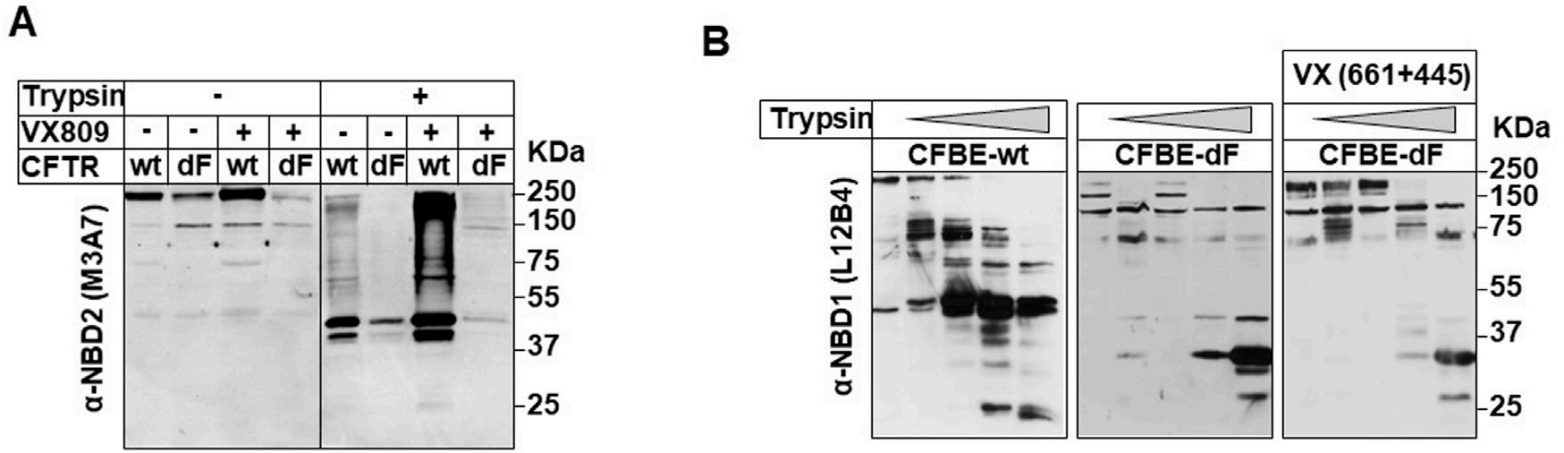
*In situ* protease susceptibility of wt-, dF508del-, and rescued F508del-CFTR. Complex glycosylated dF508-CFTR was accumulated during a 24-h rescue procedure as described in material and methods section. To substantially reduce the core-glycosylated form in wt- and rescued dF508del-CFTR expressors, cells were treated with CHX (100 μg/mL) for 3 h, and the lysates were probed with anti-CFTR mAb. Microsomes were isolated with differential centrifugation, and limited proteolysis was performed using trypsin at the following concentrations: 0, 0.05, 0.1, 0.5, and 1 mg/mL for 15 min at 4 °C. Samples (50–75 µg of protein/lane) were immunoblotted with the M3A7 **(A)**, NBD2) or L12B4 **(B)**, NBD1) anti-CFTR mAbs and visualized with ECL.

Since the half-life of core-glycosylated (immature) CFTR is approximately 2 h, cells were treated with cycloheximide (CHX) for 2.5 h to ensure the degradation of immature CFTR prior to microsome isolation. This allowed for enrichment of the mature forms of wt and rescued CFTR-F508del (Figures 6A,B).

Protease resistance of wt- and rescued CFTR-F508del to *in-situ* trypsin digestion was consistently higher following treatment with VX809 or the VX661 + VX445 combination, regardless of whether the NBD2-or NBD1-specific monoclonal antibodies (M3A7 and L12B4, respectively) were used (Figures 6A,B). These results confirm the role of correctors in stabilizing CFTR’s dynamic intramolecular interactions (Ribeiro and Gentzsch, 2021).

However, despite the corrective effects of the VX661 + VX445 combination, distinct differences in proteolysis-resistant band patterns remained (Figure 6B), indicating a persistent intrinsic folding defect.

## Discussion

Our study investigated whether restoring Cl^−^ secretion can modify the intrinsic inflammatory response of CF bronchial epithelial cells and how the structural stability of CFTR at the plasma membrane contributes to this regulatory process. Using CFBE cells expressing F508del-CFTR (CFBE-dF), we observed inflammatory profiles similar to those of parental CFBE cells, confirming previous findings regarding their intrinsic pro-inflammatory state (Kouadri et al., 2021). These cellular models are widely used in CF research to examine both inflammatory mechanisms and the pharmacological correction of CFTR (Kouadri et al., 2021; Laselva et al., 2021), providing a suitable system for dissecting the relationship between CFTR structure, function, and epithelial inflammation.

To restore CFTR function, we compared two approaches: overexpression of wt-CFTR (CFBE-wt) and treatment of CFBE-dF cells with clinically relevant CFTR modulators (VX-770, VX-809, VX-661, VX-445). As demonstrated previously for these agents, modulators act as correctors or potentiators by improving CFTR folding, trafficking, or channel activity (Okiyoneda et al., 2013). VX-809 and VX-661 stabilize specific interdomain contacts within CFTR, thereby supporting partial rescue of F508del misfolding (Okiyoneda et al., 2013). Although modulator treatment increased the appearance of mature band C and restored Cl^−^ secretion, the downstream inflammatory effects were selective. Only IL-6 and IL-17A secretion decreased, while several other cytokines increased, suggesting that modulators can trigger context-dependent pro-inflammatory signaling. Similar effects have been reported for ivacaftor and lumacaftor, which can modify cytokine release, oxidative stress, or immune cell activity independently of their effects on CFTR (Hisert et al., 2017b; Z et al., 2018; Pohl et al., 2014; Stolarczyk et al., 2020). Such responses may arise from off-target drug actions, cellular stress associated with partial rescue, or residual instability of corrected F508del-CFTR, which continues to activate inflammatory pathways (Capurro et al., 2021; Veit et al., 2020a).

These observations support the idea that recovery of channel function alone is not sufficient to normalize epithelial inflammatory signaling, a conclusion reinforced by comparable effects observed with miglustat.

In contrast, wt-CFTR expression produced a substantial decrease in IL-6, IL-8, TNFα, IL-17F, and IL-17E secretion, indicating that complete structural stability of CFTR allows broader regulation of cytokine secretion. This is consistent with reports showing that patients treated with ivacaftor exhibit reductions in some inflammatory markers (Hardisty et al., 2021), but persistent inflammatory gene expression remains detectable in individuals homozygous for F508del despite ivacaftor–lumacaftor therapy (Kopp et al., 2020). Our findings further show that VX-661 and VX-445 enhance CFTR maturation and function even under inflammatory conditions, which contrasts with previous observations suggesting that inflammation interferes with CFTR correction (Cruz et al., 2020).

Differences in the inflammatory mediators involved may account for these discrepancies, as certain cytokines and stress signals differentially influence CFTR expression, stability, or degradation (Ribeiro and Gentzsch, 2021; Prulière-Escabasse et al., 2005).

Clinical trials evaluating tezacaftor–ivacaftor and triple therapy combinations have demonstrated clear improvements in lung function and exacerbation rates (Taylor-Cousar et al., 2017; Kleizen et al., 2020). Nevertheless, inflammatory responses remain inconsistence across studies, with some reporting reduced inflammatory biomarkers (Cholon et al., 2014) and others identifying persistent activation of pathways related to IL-17 signaling, oxidative metabolism, or translational stress (Hsu et al., 2016). Importantly, VX-445 in combination with VX-661 and VX-770 restores a substantial fraction of CFTR activity (∼60–70%) but still does not fully stabilize the protein (Capurro et al., 2021), as reflected by continued ubiquitination and incomplete structural rescue. These pharmacodynamic complexities may reflect cell-type–specific effects of the modulators or differences in the extent of misfolding that VX-445 is capable of correcting (Tomati et al., 2023).

Beyond their direct effects on CFTR, modulators may act on additional epithelial processes, including mucus hydration and cellular stress pathways, which themselves shape the inflammatory environment (Harvey et al., 2022; Lussac-Sorton et al., 2023). Recent work even suggests bidirectional interactions whereby inflammation can enhance *in vitro* CFTR rescue (Ribeiro and Gentzsch, 2021), indicating that the interplay between CFTR structure and epithelial inflammatory signaling is more dynamic than previously appreciated.

Our *in-situ* protease susceptibility assays offer mechanistic insight into why modulator-corrected F508del-CFTR fails to fully attenuate inflammation. Even after treatment with VX-661/VX-445, the corrected protein did not reach the thermodynamic stability of wt-CFTR, implying that key protein–protein interactions at the apical membrane remain compromised. This is consistent with cryo-EM studies showing that VX-809/VX-661 stabilize specific intra-domain interactions in wt-CFTR (Fiedorczuk and Chen, 2022a), although such stabilization may be insufficient to fully compensate for the folding defect in the F508del variant. Clinical data also indicate that ETI therapy restores only 60%–70% of CFTR function (Middleton et al., 2019; Heijerman et al., 2019) and leaves the protein partially misfolded and thermally unstable (Froux et al., 2019). The persistence of intrinsic inflammatory activation in our model mirrors clinical observations that airway inflammation is not fully resolved under ETI therapy (Maher et al., 2025). Therefore, despite the substantial benefits of current modulators, residual structural instability of F508del-CFTR likely disrupts essential regulatory interactions required for restoring epithelial homeostasis.

This interpretation is supported by structural and proteomic studies showing that modulator-rescued CFTR continues to aberrantly associate with Hsp70/Hsp90 chaperone complexes, fails to engage stabilizing partners such as NHERF1 and syntaxin 1A, and maintains impaired coupling with other ion channels and regulatory scaffolds (Fiedorczuk and Chen, 2022b; Veit et al., 2020b). Such persistent mis-interactions likely contribute to ongoing epithelial stress and inflammation, even when channel function is partly restored. These findings highlight the need for pharmacological or genetic strategies capable of reinforcing CFTR folding, enhancing the stability of the rescued protein, and restoring its integration within broader epithelial protein networks.

Collectively, our results indicate that while CFTR modulators substantially improve Cl^−^ transport, they only partially normalize the inflammatory phenotype of CF bronchial epithelial cells. Persistent structural instability of rescued F508del-CFTR and complex cellular responses to modulators likely constrain full restoration of epithelial homeostasis. Future therapeutic strategies may therefore require combinatorial approaches targeting both CFTR folding and intrinsic inflammatory signaling to achieve more complete disease modification.

## Data Availability

The original contributions presented in the study are included in the article/Supplementary Material, further inquiries can be directed to the corresponding author.
