# Supplementary material for: Inflammatory response in CF airway epithelial cells: a comparative study of modulators and wild-type CFTR rescue
Source: Front Pharmacol. 2025 Dec 18;16:1657688. doi: 10.3389/fphar.2025.1657688 (PMC12756383; doi:10.3389/fphar.2025.1657688)
Supplement: Supplementary file 1 [file DataSheet1.pdf]

Supplementary Data

Figure S1

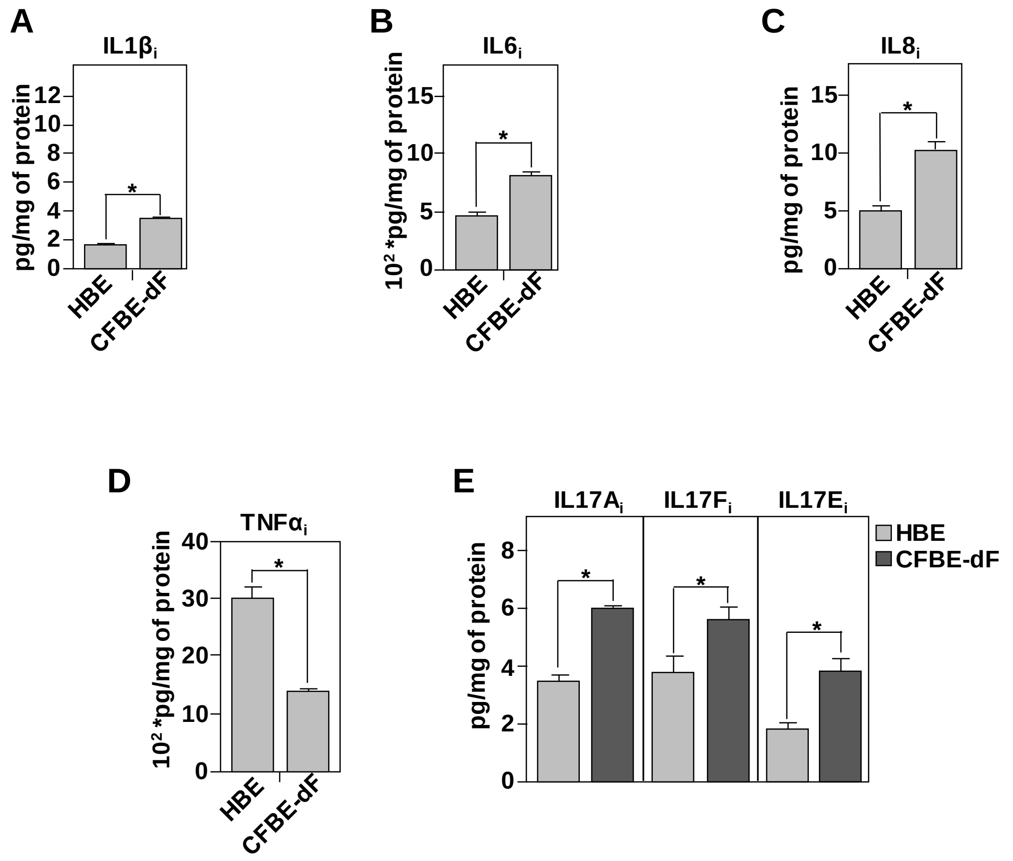

Figure S1: Inflammation profiles of HBE and CFBE-dF cells

The inflammatory profile was evaluated using a quantitative sandwich enzyme-linked immunoassay (ELISA). Measurements of IL-1β (**A**), IL-6 (**B**), IL-8 (**C**), TNFα (**D**) and IL-17 (A, F, E) (**E**) were assessed in protein extracts (i) prepared from HBE (grey box) or CFBE-dF (black box) cells. Concentrations are in pg/mg of extracted proteins. Values overwritten with asterisk are different from the control ($p < 0.05$). Data are expressed as mean ± SE ($n = 7$)

Figure S2

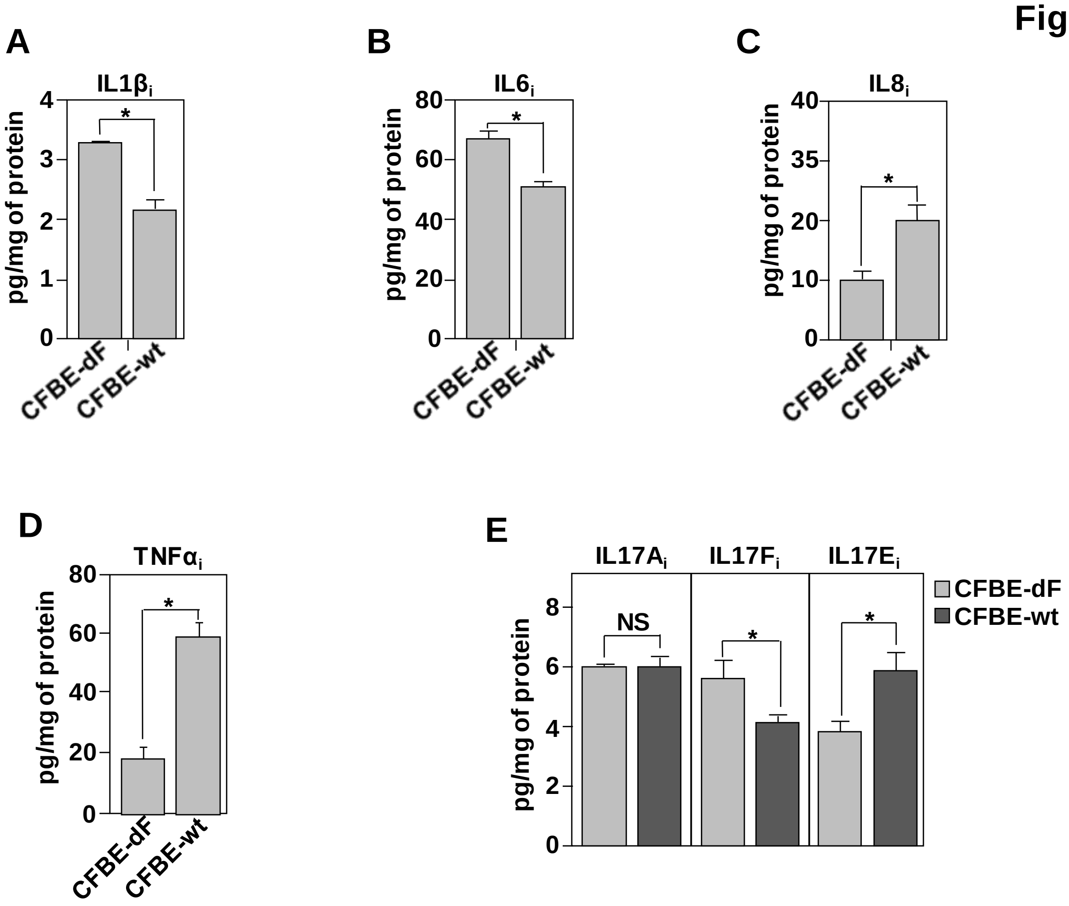

Figure S2: Comparative analysis of the production of inflammatory secretome in CFBE-dF and CFBE-wt Cells

The ELISA assay was used under the same conditions as described in Figure S1. Production (i) of IL-1β (**A**), IL-6 (**B**), IL-8 (**C**), TNFα (**D**) and IL-17 (A, F, E) (**E**) were evaluated in protein extracts prepared from CFBE-dF (grey box) or CFBE-wt (black box) cells, and expressed as pg/mg of protein extract. Values with an asterisk are significantly different from their corresponding controls ($P < 0.05$). Data are expressed as mean ± SE ($n = 7$).

Figure S3

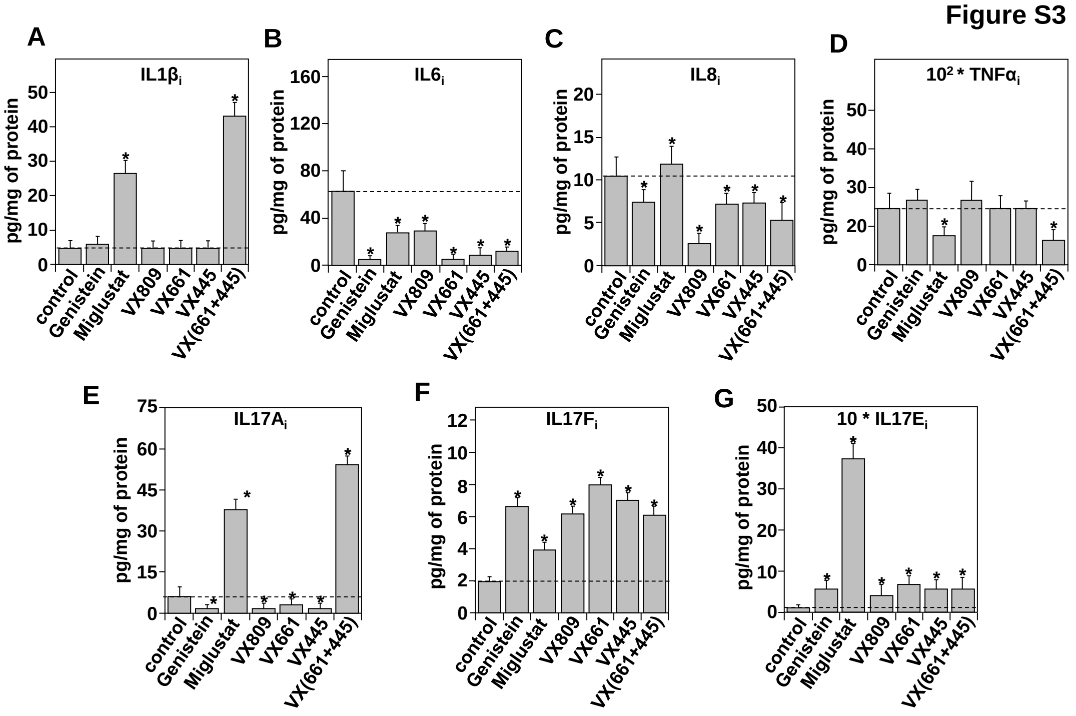

Figure S3: Impact of CFTR correctors on inflammation in CFBE-dF bronchial epithelial cells

Following 24h treatment of CFBE cells stably expressing CFTR-F508del (CFBE-dF) with the indicated correctors; genistein (50 µM), miglustat (200 µM), VX809 (3 µM), VX661 (18 µM), VX445 (3 µM) or VX445/VX661 (3 µM and 18 µM), protein extracts were prepared and tested for the production (i) of IL-1β (**A**), IL-6 (**B**), IL-8 (**C**), TNFα (**D**), IL-17A (**E**), IL-17F (**F**), and IL-17E (**G**) using ELISA assays. The results are reported in pg/mg of protein extract. Values with an asterisk are significantly different from their corresponding controls ($P < 0.05$). Data are expressed as mean ± SE (n = 7).
